# Endodontic Treatment of a Hypertaurodont Mandibular Second Molar: A Case Report

**Published:** 2011-08-15

**Authors:** Maryam Janani, Saeed Rahimi, Shahriar Shahi, Amirala Aghbali, Vahid Zand

**Affiliations:** 1. Department of Endodontics, Dental School, Tabriz University of Medical Sciences, Tabriz, Iran.; 2. Department of Endodontics, Dental and Periodontal Research Center, Dental School, Tabriz University of Medical Sciences, Tabriz, Iran.; 3. Department of Oral and Maxillofacial Pathology, Dental School, Tabriz University of Medical Sciences, Tabriz, Iran.

**Keywords:** Primary Teeth, Root Canal Treatment, Taurodontism

## Abstract

A 28-year old patient was referred for endodontic treatment of his left mandibular second molar. The patient had no history of systemic diseases and his chief complaint was spontaneous pain in left posterior region of mandible. Clinically, there was a deep caries in the left mandibular second molar. Radiographic examination of this tooth revealed a long crown containing large pulp chamber and two short roots with a apically located furcation, indicating hypertaurodontism. After coronal access preparation, five orifices were found including mesiolingual, mesiobuccal, distobuccal, mid-distal and distolingual. Subsequent to root canal preparation, a modified filling technique was used for canal obturation. After one year the treated tooth was symptom free.

## INTRODUCTION

Developmental variations of teeth are classified according to number, size, shape and structure. Taurodontism is a shape variation enclosing enlargement of the body and pulp chamber of multi-rooted tooth with the apical displacement of the pulpal floor and furcation of roots [1]. The term “taurodontism” was for the first time introduced in 1908 [2]. Taurodontism may occur in both dentitions [3], however, deciduous are more frequently affected than the permanent teeth [4]. This abnormality can be observed and diagnosed radiographically [3].

Based on furcal displacement, taurodontism has been classified into mild (hypotaurodontism), moderate (mesotaurodontism), and severe (hypertaurodontism)[1].

The prevalence of taurodontism has been reported to range between 0.25-11.3% [5][6]; the variable range is most likely due to different diagnostic criteria and racial differences [1][7][8]. Several studies evaluated the distribution of this anatomical variation in different populations [6][9][10][11][12][13][14][15]. Mandibular molars are reported to be the most commonly involved teeth [1][3][8].

The gender distribution of taurodontism appears to be equally distributed [5][6], though a study reported that males are twice as likely to be affected [11].

The etiology of taurodontism is unclear. It is believed to be subsequent to invagination in Hertwig's sheath diaphragm at an inappropriate level giving rise to a tooth with short roots, elongated body, enlarged pulp and normal dentin. Recently, a correlation has been reported between taurodontism with familial inheritance and genetic malformations [16][17]. Also, patients with labial/palatal clefts seem to be prone to taurodontism [18].

Taurodontism has been associated with syndromes such as Down, Klinefelter’s, Apert’s, oral-facial-digital (Mohr syndrome) and Tricho-dento-osseous syndrome [19]. However, the anatomic variation can occur in a normal population [6]. Some other conditions related to taurodontism are listed in Table 1.

**Table 1:** Conditions associated with taurodontism

** 1. **	Cleft lip and palate
** 2.**	Orofaciodigital syndrome
** 3.**	Tricho-dento-osseous syndrome
** 4.**	Down’s syndrome
** 5.**	Hypophosphatasia
** 6.**	Ectodermal dysplasia
** 7.**	X-Linked vitamin D-resistant rickets
** 8.**	Smith-Magenis syndrome
** 9.**	Thalassaemia major

## CASE REPORT

A 28-year old male patient was referred to Tabriz dental school for endodontic treatment of his left mandibular second molar. The patient had no history of systemic diseases; the chief complaint was spontaneous pain in left mandibular molar region.

Clinical examinations revealed deep coronal caries in the second left molar. Performing vitality tests, irreversible pulpitis was diagnosed.

**Figure 1:**
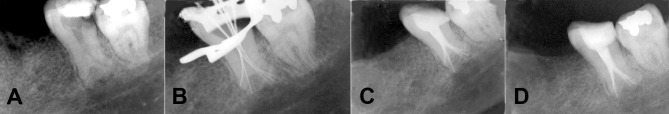
Radiographic images of mandibular second molar with taurodontism A. Preoperative radiograph, B. working length determination, C. final image, D. one-year follow up.

Radiographic image revealed a large pulp chamber with an elongated body of tooth, shortened roots and furcation located apically (Figure 1A) [20][9][10][6].

The patient received a block injection of lidocaine with 1:100,000 epinephrine. After access cavity preparation, the tooth was isolated with rubber dam. The pulp chamber was large and the floor of the pulp was undetermined. Therefore, an endodontic microscope was used for tracking canal orifices.

In the furcation area of chamber floor five orifices were found including mesiolingual (ML), mesiobuccal (MB), distobuccal (DB), mid-distal and distolingual (DL).

Working length was determined with K-file #15 (Figure 1B) and confirmed with an electronic apex finder (J. Morita Mfg Corp, Kyoto, Japan). MB, ML and MD were instrumented up to #35 and DB and DL were instrumented up to #30. Canals were irrigated with 2.5% sodium hypochlorite (NaOCl). After final preparation, canals were dried with paper points and a modified filling technique was used for canal obturation. First, AH26 sealer was used; the sealer was placed on root walls with a lentulo spiral. Subsequently, sealer-coated gutta-percha cones were placed to the working length. A #25 spreader was placed next to the master cone in a vertical and rotary manner. Then the spreader was pulled out and #20 accessory cones were placed in the canals. The remaining coronal portions of canals and the pulp chamber were filled with warm gutta-percha technique (warm vertical compaction) (Figure 1C). The access cavity was sealed with Cavit temporary filling material and the patient was referred for final restorative treatment. After one year the tooth was asymptomatic (Figure 1D).

## DISCUSSION

Compared to other molars, mandibular second molar teeth have more variations in canal configuration, for example c-shaped canals [19]. Taurodontism is an anomaly in tooth shape characterized by elongated pulp chamber and shortened tooth roots [1]. Recent studies have shown that this anomaly is not rare and occurs within 0.25-11.3% of the population [5][6]. This anomaly can be found with or without other syndromes. Some syndromic anomalies like Down, Klinefelter’s, Apert’s, oral-facial-digital (Mohr syndrome) and Tricho-dento-osseous syndrome are related to this anomaly [20]; also it has shown high prevalence in accompany with labial/palatal clefts [18]. However, this anomaly is also common in healthy populations [6].

Shaw introduced a classification for the degree of taurodontism [21]; our case can be classified as hypertaurodont with five orifices and five separate root canals. Similar published cases are rare; only one case of mandibular second molar with five orifices has been reported and only three were treated up to the apex [22]. In this case we tried to prepare all five canals and fill them with recommended modified technique [23]. Because of the difficulty in localization and preparation of the canals in these teeth, magnification proved to be essential.

Using 2.5% sodium hypochlorite has been recommended by Widerman and Setene for dissolving the remaining pulp tissues [24]. Because of the complexity of irregular root canal system in taurodonts, sufficient instrumentation may be impossible; therefore, sodium hypochlorite can improve root canal cleaning. We used the recommended modified obturation technique; i.e. lateral compaction apically and warm vertical compaction or injection coronally [23].

## CONCLUSION

Endodontic treatment of taurodont teeth is often time-consuming and challenging. Careful evalu-ation for additional canals is essential due to potential of taurodontism in abnormal root canal system.
